# 2-Isobutyl-2-phosphabicyclo­[3.3.1]nonane 2-selenide

**DOI:** 10.1107/S1600536809005492

**Published:** 2009-02-21

**Authors:** Peter N. Bungu, Stefanus Otto

**Affiliations:** a Sasol Technology Research & Development, 1 Klasie Havenga Road, Sasolburg 1947, South Africa; b Department of Chemistry, University of the Free State, PO Box 339, Bloemfontein 9300, South Africa

## Abstract

The title compound, C_12_H_23_PSe, represents the first structure of a phosphine containing the bicyclic 2-phospha­bicyclo­[3.3.1]nonane (VCH) unit. It contains two chiral centres per mol­ecule which can be either *R*,*R*- or *S*,*S* and crystallizes as a centrosymmetric, racemic micture of the enanti­omers. The P—Se bond distance of 2.1360 (16) Å is typical for these compounds. The Tolman cone angle (2.28 Å from P) was calculated as 163°, and the effective cone angle (using the crystallographically determined P—Se bond distance) is 168°.

## Related literature

For the synthesis of phosphine selenides, see: Otto *et al.* (2005 ▶). For the evaluation of ligand electronic properties, see: Allen & Taylor (1982 ▶); Bungu & Otto (2007*b* ▶); Muller *et al.* (2008 ▶); Otto & Roodt (2004 ▶); Roodt & Steyn (2000 ▶). For the application of bicyclic ligands in catalysis, see: Bungu & Otto (2007*a* ▶); Crause *et al.* (2003 ▶); Dwyer *et al.* (2004 ▶); Steynberg *et al.* (2003 ▶); Van Winkle *et al.* (1969 ▶). For information on cone angles, see: Tolman (1977 ▶); Otto (2001 ▶).
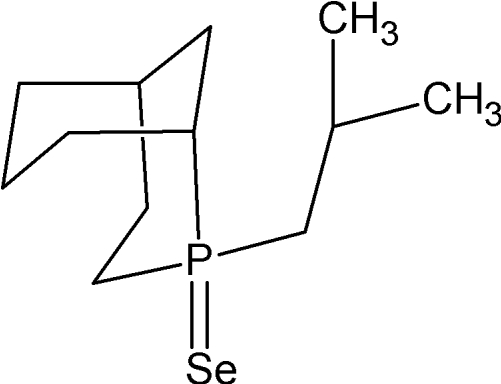


## Experimental

### 

#### Crystal data

C_12_H_23_PSe
*M* *_r_* = 277.23
Monoclinic, 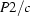

*a* = 10.763 (2) Å
*b* = 7.2540 (15) Å
*c* = 17.530 (4) Å
β = 97.93 (3)°
*V* = 1355.6 (5) Å^3^
*Z* = 4
Mo *K*α radiation
μ = 2.85 mm^−1^
*T* = 293 K
0.14 × 0.12 × 0.08 mm

#### Data collection

Bruker X8 APEXII 4K KappaCCD diffractometer
Absorption correction: multi-scan (*SADABS*; Bruker, 2008 ▶) *T*_min_ = 0.691, *T*_max_ = 0.804
9132 measured reflections
3366 independent reflections
1658 reflections with *I* > 2σ(*I*)
*R*_int_ = 0.062

#### Refinement

*R*[*F* ^2^ > 2σ(*F* ^2^)] = 0.059
*wR*(*F* ^2^) = 0.181
*S* = 1.02
3366 reflections
129 parameters
H-atom parameters constrained
Δρ_max_ = 0.58 e Å^−3^
Δρ_min_ = −0.58 e Å^−3^

### 

Data collection: *APEX2* (Bruker, 2008 ▶); cell refinement: *SAINT-Plus* (Bruker, 2004 ▶); data reduction: *SAINT-Plus* and *XPREP* (Bruker, 2004 ▶); program(s) used to solve structure: *SHELXS97* (Sheldrick, 2008 ▶); program(s) used to refine structure: *SHELXL97* (Sheldrick, 2008 ▶); molecular graphics: *DIAMOND* (Brandenburg & Berndt, 2001 ▶); software used to prepare material for publication: *SHELXL97*.

## Supplementary Material

Crystal structure: contains datablocks global, I. DOI: 10.1107/S1600536809005492/hb2909sup1.cif

Structure factors: contains datablocks I. DOI: 10.1107/S1600536809005492/hb2909Isup2.hkl

Additional supplementary materials:  crystallographic information; 3D view; checkCIF report

## Figures and Tables

**Table 1:** X-ray and spectroscopic data (Å, Hz) for selected phosphine selenides.

P	Se—P	^1^*J*_Se—P_
PMe_3_^i^	2.111 (3)	684
PCy_3_^ii^	2.108 (1)	676
VCH-^i^Bu^iii^	2.1360 (16)	672, 687
Phoban-Ph^iv^	2.1090 (9)	689, 717
PPhCy_2_^v^	2.1260 (8)	701
P(*o*-Tol)_3_^vi^	2.116 (5)	708
PPh_2_Cy^v^	2.111 (2)	725
PPh_3_^vii^	2.106 (1)	733
P(NMe_2_)_3_^viii^	2.120 (1)	797

Notes: (i) Cogne *et al.* (1980 ▶); (ii) Davies *et al.* (1991 ▶); (iii) this work; (iv) Bungu & Otto (2007*b* ▶); (v) Muller et al. (2008 ▶); (vi) Cameron & Dahlen (1975 ▶); (vii) Codding & Kerr (1979 ▶); (viii) Rømming & Songstad (1979 ▶).

**Fig. 1.**
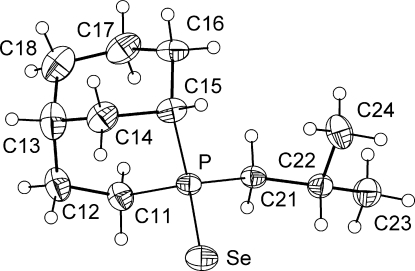
Molecular diagram of (I) showing 30% displacement ellipsoids.

## supplementary crystallographic information

### Comment

It is well established that the steric and electronic properties of phosphine ligands have a major influence on the chemistry of its metal species. Sevaral methods are used to quantify the electronic characteristics of phosphines, including NMR measurements of first order Pt—P, Rh—P, Se—P and P—BH_3_ coupling constants (Allen & Taylor, 1982 and Roodt & Steyn, 2000) and measuring of CO stretching frequencies in complexes such as [Ni(*L*)(CO)_3_] (Tolman, 1977) or *trans*-[RhCl(CO)(*L*)_2_] (Otto & Roodt, 2004).

Phosphine ligands containing bicyclic substituents have been shown to add significant benefits to the catalytic performance of several homogeneously catalysed systems (Bungu & Otto, 2007*a*). The best known example is the 9-phosphabicyclo[3.3.1]nonane and 9-phosphabicyclo[4.2.1]nonane mixture of isomers (Phoban family of ligands) patented by Shell (Van Winkle *et al.*, 1969) for modified cobalt hydroformylation. Sasol has reported on the use of bicyclic phosphines derived from (*R*)-(+)-Limonene (Lim family) (Crause *et al.*, 2003, Dwyer *et al.*, 2004) and vinylcyclohexene (VCH family) (Steynberg *et al.*, 2003) for similar applications.

After a convenient synthetic protocol for phosphine selenides were developed (Otto *et al.*, 2005) we extensively used the Se—P coupling constants for the quantification of electronic properties of phosphine ligands (Bungu & Otto, 2007*b*). Smaller values for the coupling constants correspond with ligands of higher basicity (more electron donating). We now report the synthesis and crystallographic characterization of the title compound, (I), 2-isobutyl-2-phosphabicyclo[3.3.1]nonane 2-selenide (VCH-^i^Bu) which represents the first crystal structure of a VCH family member.

Compound (I) crystallizes in the monoclinic space group P2/c and consists of the VCH backbone, the iso-butyl side chain and the selenium atom coordinated to phosphorus in a tetrahedral fashion. The compound contains two chiral centres on the VCH backbone (C13 and C15) which can be *R*,*R*- or S,*S* (as in the arbitrarily chosen asymmetric molecule; Fig. 1) and it crystallizes as a racemic mixture on account of the centrosymmetric space group. The P atom could also be considered as chiral based on the four different substituents. All bond distances and angles are within normal ranges. Even though larger Se—P coupling constants are indicative of more effective s-orbital overlap no clear trends are evident in the Se—P bond distances.

The packing in the unit cell is governed by van der Waals forces alone since no pertinent intramolecular interactions were evident. The Tolman- (2.28 Å from P) and effective cone angles (using the crystallographically determined Se—P bond distance) were calculated (Otto, 2001) resulting in values of 163 and 168° respectively.

### Experimental

2-Isobutyl-2-phosphabicyclo[3.3.1]nonane was generously supplied by Cytec; it exists as two stereo isomers in close to equal quantities. ^31^P (CDCl_3_): -36.23 and -35.22 p.p.m..

The title compound was prepared according to the procedure described previously (Bungu & Otto, 2007*b*), colourless blocks of (I) were obtained by evaporation of a dichloromethane solution. ^31^P (CDCl_3_): 29.72 p.p.m. (^1^*J*_Se—P_ = 684 Hz) and 29.88 p.p.m. (^1^*J*_Se—P_ = 670 Hz).

### Refinement

The H atoms were placed in geometrically idealized positions (CH = 0.98, CH_2_ = 0.97 and CH_3_ = 0.96 Å) and constrained to ride on their parent atoms with *U*_iso_(H) = 1.2*U*_eq_(C) for CH and CH_2_ and *U*_iso_(H) = 1.5*U*_eq_(C) for CH_3_.

### Crystal data

**Table:**

C_12_H_23_PSe	*F*(000) = 576
*M**_r_* = 277.23	*D*_x_ = 1.358 Mg m^−^^3^
Monoclinic, *P*2/*c*	Mo *K*α radiation, λ = 0.71073 Å
*a* = 10.763 (2) Å	Cell parameters from 1002 reflections
*b* = 7.2540 (15) Å	θ = 2.8–25.7°
*c* = 17.530 (4) Å	µ = 2.85 mm^−^^1^
β = 97.93 (3)°	*T* = 293 K
*V* = 1355.6 (5) Å^3^	Block, colourless
*Z* = 4	0.14 × 0.12 × 0.08 mm

### Data collection

**Table:**

Bruker X8 APEXII 4K KappaCCD diffractometer	3366 independent reflections
Radiation source: fine-focus sealed tube	1658 reflections with *I* > 2σ(*I*)
graphite	*R*_int_ = 0.062
Detector resolution: 512 pixels mm^-1^	θ_max_ = 28.3°, θ_min_ = 1.9°
φ and ω scans	*h* = −11→14
Absorption correction: multi-scan (*SADABS*; Bruker, 2008)	*k* = −8→9
*T*_min_ = 0.691, *T*_max_ = 0.804	*l* = −23→23
9132 measured reflections	

### Refinement

**Table:**

Refinement on *F*^2^	Primary atom site location: structure-invariant direct methods
Least-squares matrix: full	Secondary atom site location: difference Fourier map
*R*[*F*^2^ > 2σ(*F*^2^)] = 0.059	Hydrogen site location: inferred from neighbouring sites
*wR*(*F*^2^) = 0.181	H-atom parameters constrained
*S* = 1.02	*w* = 1/[σ^2^(*F*_o_^2^) + (0.0816*P*)^2^ + 0.9034*P*] where *P* = (*F*_o_^2^ + 2*F*_c_^2^)/3
3366 reflections	(Δ/σ)_max_ < 0.001
129 parameters	Δρ_max_ = 0.58 e Å^−^^3^
0 restraints	Δρ_min_ = −0.58 e Å^−^^3^

### Special details

**Table:**

Experimental. The intensity data were collected on a Bruker X8 ApexII 4 K Kappa CCD diffractometer using an exposure time of 40 s/frame with a frame width of 0.3°; a total of 1315 frames were collected.The crystals were of poor quality and were, as a precautionary measure, covered with Canada balsam. Consequently some intensity in the reflextions were sacrificed and the completeness is somewhat low at high angles.
Geometry. All e.s.d.'s (except the e.s.d. in the dihedral angle between two l.s. planes) are estimated using the full covariance matrix. The cell e.s.d.'s are taken into account individually in the estimation of e.s.d.'s in distances, angles and torsion angles; correlations between e.s.d.'s in cell parameters are only used when they are defined by crystal symmetry. An approximate (isotropic) treatment of cell e.s.d.'s is used for estimating e.s.d.'s involving l.s. planes.
Refinement. Refinement of *F*^2^ against ALL reflections. The weighted *R*-factor *wR* and goodness of fit *S* are based on *F*^2^, conventional *R*-factors *R* are based on *F*, with *F* set to zero for negative *F*^2^. The threshold expression of *F*^2^ > σ(*F*^2^) is used only for calculating *R*-factors(gt) *etc*. and is not relevant to the choice of reflections for refinement. *R*-factors based on *F*^2^ are statistically about twice as large as those based on *F*, and *R*- factors based on ALL data will be even larger.

### Fractional atomic coordinates and isotropic or equivalent isotropic displacement parameters (Å^2^)

**Table:**

	*x*	*y*	*z*	*U*_iso_*/*U*_eq_	
P	0.21847 (13)	−0.0934 (2)	−0.03355 (8)	0.0418 (4)	
Se	0.23308 (6)	0.16679 (9)	0.02405 (4)	0.0633 (3)	
C11	0.1179 (6)	−0.0851 (9)	−0.1269 (3)	0.0588 (16)	
H11A	0.0957	−0.2100	−0.1433	0.071*	
H11B	0.0410	−0.0206	−0.1207	0.071*	
C12	0.1797 (6)	0.0105 (11)	−0.1899 (4)	0.0712 (19)	
H12A	0.1870	0.1411	−0.1780	0.085*	
H12B	0.1251	−0.0022	−0.2384	0.085*	
C13	0.3099 (7)	−0.0640 (12)	−0.1997 (4)	0.078 (2)	
H13	0.3411	0.0092	−0.2401	0.094*	
C14	0.3999 (6)	−0.0340 (10)	−0.1255 (4)	0.0699 (19)	
H14A	0.3940	0.0930	−0.1090	0.084*	
H14B	0.4852	−0.0554	−0.1353	0.084*	
C15	0.3697 (5)	−0.1675 (8)	−0.0582 (4)	0.0528 (15)	
H15	0.4336	−0.1494	−0.0133	0.063*	
C16	0.3785 (6)	−0.3676 (8)	−0.0870 (4)	0.0610 (17)	
H16A	0.4652	−0.3938	−0.0924	0.073*	
H16B	0.3544	−0.4505	−0.0481	0.073*	
C17	0.2984 (7)	−0.4081 (11)	−0.1622 (4)	0.082 (2)	
H17A	0.2112	−0.4131	−0.1538	0.099*	
H17B	0.3208	−0.5285	−0.1802	0.099*	
C18	0.3119 (8)	−0.2667 (14)	−0.2240 (5)	0.091 (2)	
H18A	0.2446	−0.2859	−0.2661	0.110*	
H18B	0.3903	−0.2900	−0.2438	0.110*	
C21	0.1449 (5)	−0.2707 (7)	0.0190 (3)	0.0452 (13)	
H21A	0.0547	−0.2558	0.0076	0.054*	
H21B	0.1655	−0.3899	−0.0010	0.054*	
C22	0.1795 (5)	−0.2755 (8)	0.1068 (3)	0.0490 (14)	
H22	0.1706	−0.1505	0.1266	0.059*	
C23	0.0893 (6)	−0.4026 (10)	0.1427 (4)	0.0680 (18)	
H23A	0.0046	−0.3618	0.1277	0.102*	
H23B	0.1087	−0.3991	0.1978	0.102*	
H23C	0.0981	−0.5265	0.1250	0.102*	
C24	0.3154 (6)	−0.3372 (10)	0.1303 (4)	0.0708 (19)	
H24A	0.3258	−0.4603	0.1121	0.106*	
H24B	0.3350	−0.3345	0.1854	0.106*	
H24C	0.3707	−0.2554	0.1080	0.106*	

### Atomic displacement parameters (Å^2^)

**Table:**

	*U*^11^	*U*^22^	*U*^33^	*U*^12^	*U*^13^	*U*^23^
P	0.0394 (8)	0.0373 (7)	0.0482 (8)	−0.0002 (6)	0.0044 (6)	−0.0054 (6)
Se	0.0709 (5)	0.0383 (4)	0.0804 (5)	−0.0015 (3)	0.0100 (3)	−0.0152 (3)
C11	0.056 (4)	0.061 (4)	0.057 (4)	0.000 (3)	0.002 (3)	0.007 (3)
C12	0.062 (4)	0.085 (5)	0.063 (4)	−0.003 (4)	−0.001 (3)	0.022 (4)
C13	0.067 (5)	0.105 (7)	0.060 (4)	−0.018 (4)	0.001 (3)	0.017 (4)
C14	0.062 (4)	0.074 (5)	0.077 (5)	−0.013 (3)	0.020 (4)	−0.006 (4)
C15	0.039 (3)	0.054 (4)	0.065 (4)	−0.005 (3)	0.006 (3)	−0.014 (3)
C16	0.055 (4)	0.047 (4)	0.083 (5)	0.008 (3)	0.018 (3)	−0.010 (3)
C17	0.091 (6)	0.080 (5)	0.078 (5)	−0.010 (4)	0.019 (4)	−0.032 (4)
C18	0.093 (6)	0.108 (7)	0.075 (5)	−0.014 (5)	0.020 (4)	−0.018 (5)
C21	0.047 (3)	0.036 (3)	0.052 (3)	0.001 (2)	0.008 (3)	−0.003 (2)
C22	0.056 (4)	0.042 (3)	0.049 (3)	0.000 (3)	0.009 (3)	−0.004 (3)
C23	0.073 (5)	0.063 (4)	0.073 (4)	−0.001 (4)	0.027 (4)	0.010 (3)
C24	0.068 (4)	0.078 (5)	0.062 (4)	−0.008 (4)	−0.009 (3)	0.013 (4)

### Geometric parameters (Å, °)

**Table:**

P—C15	1.822 (6)	C16—H16A	0.9700
P—C21	1.825 (6)	C16—H16B	0.9700
P—C11	1.835 (6)	C17—C18	1.514 (12)
P—Se	2.1360 (16)	C17—H17A	0.9700
C11—C12	1.531 (9)	C17—H17B	0.9700
C11—H11A	0.9700	C18—H18A	0.9700
C11—H11B	0.9700	C18—H18B	0.9700
C12—C13	1.534 (10)	C21—C22	1.533 (8)
C12—H12A	0.9700	C21—H21A	0.9700
C12—H12B	0.9700	C21—H21B	0.9700
C13—C14	1.527 (9)	C22—C24	1.530 (9)
C13—C18	1.532 (12)	C22—C23	1.536 (8)
C13—H13	0.9800	C22—H22	0.9800
C14—C15	1.594 (9)	C23—H23A	0.9600
C14—H14A	0.9700	C23—H23B	0.9600
C14—H14B	0.9700	C23—H23C	0.9600
C15—C16	1.544 (8)	C24—H24A	0.9600
C15—H15	0.9800	C24—H24B	0.9600
C16—C17	1.501 (9)	C24—H24C	0.9600
			
C15—P—C21	112.0 (3)	C17—C16—H16B	108.6
C15—P—C11	103.6 (3)	C15—C16—H16B	108.6
C21—P—C11	103.3 (3)	H16A—C16—H16B	107.6
C15—P—Se	111.27 (19)	C16—C17—C18	113.3 (6)
C21—P—Se	113.12 (19)	C16—C17—H17A	108.9
C11—P—Se	112.9 (2)	C18—C17—H17A	108.9
C12—C11—P	113.3 (4)	C16—C17—H17B	108.9
C12—C11—H11A	108.9	C18—C17—H17B	108.9
P—C11—H11A	108.9	H17A—C17—H17B	107.7
C12—C11—H11B	108.9	C17—C18—C13	116.4 (6)
P—C11—H11B	108.9	C17—C18—H18A	108.2
H11A—C11—H11B	107.7	C13—C18—H18A	108.2
C11—C12—C13	114.6 (6)	C17—C18—H18B	108.2
C11—C12—H12A	108.6	C13—C18—H18B	108.2
C13—C12—H12A	108.6	H18A—C18—H18B	107.3
C11—C12—H12B	108.6	C22—C21—P	117.4 (4)
C13—C12—H12B	108.6	C22—C21—H21A	107.9
H12A—C12—H12B	107.6	P—C21—H21A	107.9
C14—C13—C18	110.0 (7)	C22—C21—H21B	107.9
C14—C13—C12	109.6 (6)	P—C21—H21B	107.9
C18—C13—C12	114.7 (6)	H21A—C21—H21B	107.2
C14—C13—H13	107.4	C24—C22—C21	111.5 (5)
C18—C13—H13	107.4	C24—C22—C23	110.5 (5)
C12—C13—H13	107.4	C21—C22—C23	110.1 (5)
C13—C14—C15	112.0 (5)	C24—C22—H22	108.2
C13—C14—H14A	109.2	C21—C22—H22	108.2
C15—C14—H14A	109.2	C23—C22—H22	108.2
C13—C14—H14B	109.2	C22—C23—H23A	109.5
C15—C14—H14B	109.2	C22—C23—H23B	109.5
H14A—C14—H14B	107.9	H23A—C23—H23B	109.5
C16—C15—C14	107.5 (5)	C22—C23—H23C	109.5
C16—C15—P	116.8 (4)	H23A—C23—H23C	109.5
C14—C15—P	106.0 (4)	H23B—C23—H23C	109.5
C16—C15—H15	108.7	C22—C24—H24A	109.5
C14—C15—H15	108.7	C22—C24—H24B	109.5
P—C15—H15	108.7	H24A—C24—H24B	109.5
C17—C16—C15	114.7 (6)	C22—C24—H24C	109.5
C17—C16—H16A	108.6	H24A—C24—H24C	109.5
C15—C16—H16A	108.6	H24B—C24—H24C	109.5
			
C15—P—C11—C12	−45.9 (6)	C11—P—C15—C14	50.9 (4)
C21—P—C11—C12	−162.9 (5)	Se—P—C15—C14	−70.7 (4)
Se—P—C11—C12	74.6 (5)	C14—C15—C16—C17	−54.2 (7)
P—C11—C12—C13	52.2 (8)	P—C15—C16—C17	64.7 (7)
C11—C12—C13—C14	−62.0 (8)	C15—C16—C17—C18	48.6 (9)
C11—C12—C13—C18	62.3 (8)	C16—C17—C18—C13	−45.1 (10)
C18—C13—C14—C15	−55.5 (8)	C14—C13—C18—C17	48.8 (9)
C12—C13—C14—C15	71.5 (8)	C12—C13—C18—C17	−75.3 (8)
C13—C14—C15—C16	58.2 (7)	C15—P—C21—C22	87.0 (5)
C13—C14—C15—P	−67.5 (6)	C11—P—C21—C22	−162.1 (4)
C21—P—C15—C16	41.8 (6)	Se—P—C21—C22	−39.8 (5)
C11—P—C15—C16	−68.9 (5)	P—C21—C22—C24	−69.3 (6)
Se—P—C15—C16	169.6 (4)	P—C21—C22—C23	167.7 (4)
C21—P—C15—C14	161.6 (4)		

### Table 1 X-ray and spectroscopic data (Å, Hz) for selected phosphine selenides.

**Table:**

P	Se—P	^1^*J*_Se—P_
PMe_3_^i^	2.111 (3)	684
PCy_3_^ii^	2.108 (1)	676
VCH-^i^Bu^iii^	2.1360 (16)	672,687
Phoban-Ph^iv^	2.1090 (9)	689,717
PPhCy_2_^v^	2.1260 (8)	701
P(o-Tol)_3_^vi^	2.116 (5)	708
PPh_2_Cy^v^	2.111 (2)	725
PPh_3_^vii^	2.106 (1)	733
P(NMe_2_)_3_^viii^	2.120 (1)	797

Notes: (i) Cogne *et al.* (1980); (ii) Davies *et al.* (1991); (iii) this work; (iv) Bungu & Otto (2007*b*); (v) Muller *et al.* (2008); (vi) Cameron & Dahlen (1975); (vii) Codding & Kerr (1979); (viii) Rømming & Songstad (1979).

## References

[bb1] Allen, D. W. & Taylor, B. F. J. (1982). *Chem. Soc. Dalton Trans.* pp. 51–54.

[bb2] Brandenburg, K. & Berndt, M. (2001). *DIAMOND* Crystal Impact GbR, Bonn, Germany.

[bb3] Bruker (2004). *SAINT-Plus* (including *XPREP*). Bruker AXS Inc., Madison, Wisconsin, USA.

[bb4] Bruker (2008). *APEX2* and *SADABS* Bruker AXS Inc., Madison, Wisconsin, USA.

[bb5] Bungu, P. N. & Otto, S. (2007*a*). *Dalton Trans.* pp. 2876–2884. 10.1039/b702709e 17607402

[bb6] Bungu, P. N. & Otto, S. (2007*b*). *J. Organomet. Chem.* **692**, 3370–3379.

[bb7] Cameron, T. S. & Dahlen, B. (1975). *J. Chem. Soc. Perkin Trans. 2*, pp. 1737–1751.

[bb8] Codding, P. W. & Kerr, K. A. (1979). *Acta Cryst.* B**35**, 1261–1263.

[bb9] Cogne, A., Grant, A., Laugier, J., Robert, J. B. & Wiesenfield, L. (1980). *J. Am. Chem. Soc.* **102**, 2238–2242.

[bb10] Crause, C., Bennie, L., Damoense, L., Dwyer, C. L., Grove, C., Grimmer, N., Janse van Rensburg, W., Kirk, M. M., Mokheseng, K. M., Otto, S. & Steynberg, P. J. (2003). *Dalton Trans.* pp. 2036–2042.

[bb11] Davies, J. A., Dutremez, S. & Pinkerton, A. A. (1991). *Inorg. Chem.* **30**, 2380–2387.

[bb12] Dwyer, C., Assumption, H., Coetzee, J., Crause, C., Damoense, L. & Kirk, M. M. (2004). *Coord. Chem. Rev.* **248**, 653–670.

[bb13] Muller, A., Otto, S. & Roodt, A. (2008). *Dalton Trans.* pp. 650–657. 10.1039/b712782k 18217121

[bb14] Otto, S. (2001). *Acta Cryst.* C**57**, 793–795. 10.1107/s0108270101006035 11443242

[bb15] Otto, S., Ionescu, A. & Roodt, A. (2005). *J. Organomet. Chem.* **690**, 4337–4342.

[bb16] Otto, S. & Roodt, A. (2004). *Inorg. Chim. Acta*, **357**, 1–10.

[bb17] Rømming, C. & Songstad, J. (1979). *Acta Chem. Scand. Ser. A*, **33**, 187–197.

[bb18] Roodt, A. & Steyn, G. J. J. (2000). * Recent Research Developments in Inorganic Chemistry*, Vol. 2, edited by S. G. Pandalai, pp. 1–23. Trivandrum: Transworld Research Network.

[bb19] Sheldrick, G. M. (2008). *Acta Cryst.* A**64**, 112–122. 10.1107/S0108767307043930 18156677

[bb20] Steynberg, P. J., Van Rensburg, H., Grove, J. J. C., Otto, S. & Crause, C. (2003). Int. Appl. WO, 2003068719, A2.

[bb21] Tolman, C. A. (1977). *Chem. Rev.* **77**, 313–348.

[bb22] Van Winkle, J. L., Lorenzo, S., Morris, R. C. & Mason, R. F. (1969). US Patent No. 3 420 898.

